# Epigenetic mechanisms in traumatic brain injury: a focus on astrocytes and therapeutic implications

**DOI:** 10.1186/s12974-026-03974-1

**Published:** 2026-07-22

**Authors:** Lulin Li, Alyssa M. Bonfoey, Bryan Sun, Odette A. Harris, Jian Luo

**Affiliations:** 1 Palo Alto Veterans Institute for Research, VA Palo Alto Health Care System, Palo Alto, CA 94304 USA; 2 Department of Neurosurgery, Stanford University School of Medicine, Stanford, CA 94305 USA; 3 Polytrauma System of Care, VA Palo Alto Health Care System, Palo Alto, CA 94304 USA

**Keywords:** Traumatic brain injury, Epigenetic remodeling, Astrocytes, Reactive astrogliosis, Epigenetic memory, Neuroinflammation, Neurodegeneration

## Abstract

Traumatic brain injury (TBI) triggers complex molecular and cellular responses that persist well beyond the initial injury. Neuroinflammation is a prominent feature of the post-injury response and a significant contributor to progressive neuronal dysfunction and increased risk of chronic neurodegeneration. Epigenetic mechanisms have emerged as important regulators of the injury response. Astrocytes are increasingly recognized as key mediators of this epigenetic regulation. Injury-induced epigenetic reprogramming in astrocytes may perpetuate maladaptive inflammatory and metabolic states, thereby increasing vulnerability to delayed neurodegeneration. Preclinical studies suggest that pharmacologic or genetic modulation of epigenetic regulators can improve outcomes after experimental TBI. However, clinical translation remains constrained by limited cell-type specificity and potential off-target effects. This review evaluates current evidence on epigenetic regulation after TBI, with an emphasis on astrocyte-centered mechanisms linking acute injury to chronic pathology. We further discuss key translational challenges and highlight emerging precision strategies aimed at cell-type-specific, temporally controlled epigenetic modulation to mitigate long-term neurological consequences.

## Introduction

Traumatic brain injury (TBI) is a leading cause of death and long-term disability and a major risk factor for chronic neurodegenerative diseases [1]. TBI pathophysiology involves a primary injury resulting from the initial mechanical impact, followed by a secondary injury characterized by a complex cascade of molecular and cellular disturbances. These secondary processes include, for example, excitotoxicity, oxidative stress, neuroinflammation, blood–brain barrier (BBB) disruption, synaptic dysfunction, and progressive cell death [2–5]. Together, these processes drive the transition from acute tissue damage to chronic neurodegeneration and affect long-term outcomes [2–5]. Epidemiological studies indicate that TBI, particularly repeated mild injury, increases the risk for neurodegenerative diseases, such as Alzheimer’s disease, Parkinson’s disease, and chronic traumatic encephalopathy [6, 7]. Chronic neuroinflammation, which can last for years after injury, likely contributes to this elevated risk [7–9].

While traditional investigations have focused on signaling pathways and transcriptional changes following TBI [8], epigenetic regulation provides an underlying mechanism for sustained injury-induced phenotypic changes [10, 11]. Epigenetic mechanisms, including DNA methylation, histone modifications, and non-coding RNA (ncRNA) regulation, regulate gene expression and provide a molecular basis for the long-term persistence of post-injury cellular states. Importantly, these responses are stage-dependent [11] and are major determinants of recovery [12].

After TBI, epigenetic alterations occur across multiple brain cell types, including neurons, glia, and vascular-associated cells [11]. Among these, astrocytes are uniquely positioned to integrate injury signals, as they are long-lived, largely post-mitotic cells that play central roles in maintaining brain homeostasis [13]. Astrocytes also exhibit remarkable functional plasticity, transitioning between diverse reactive states in response to injury and disease [14, 15]. Emerging evidence indicates that these state transitions are regulated, at least in part, by chromatin remodeling and epigenetic mechanisms [16–18]. These characteristics make astrocytes particularly compelling candidates for sustained injury-induced epigenetic remodeling and long-term transcriptional reprogramming. Therefore, astrocyte-specific epigenetic mechanisms may thus represent a critical link between acute injury and the chronic neuroinflammation and neurodegeneration that follow.

Several recent reviews have summarized epigenetic regulation following TBI, including mechanisms, temporal dynamics, and therapeutic interventions [10, 11, 19–21]. Other reviews have highlighted the broader importance of astrocyte epigenetics across neurological disorders [17]. However, the role of astrocyte-specific epigenetic remodeling in linking acute TBI to chronic neuroinflammation and neurodegenerative vulnerability has not been comprehensively examined. Here, we review current evidence for astrocyte epigenetic remodeling after TBI, discuss the emerging concept of astrocytic epigenetic memory, evaluate its supporting evidence and limitations, and outline key experimental priorities needed to establish causal relationships and therapeutic relevance.

## Core epigenetic mechanisms activated by TBI

**Fig.1:**
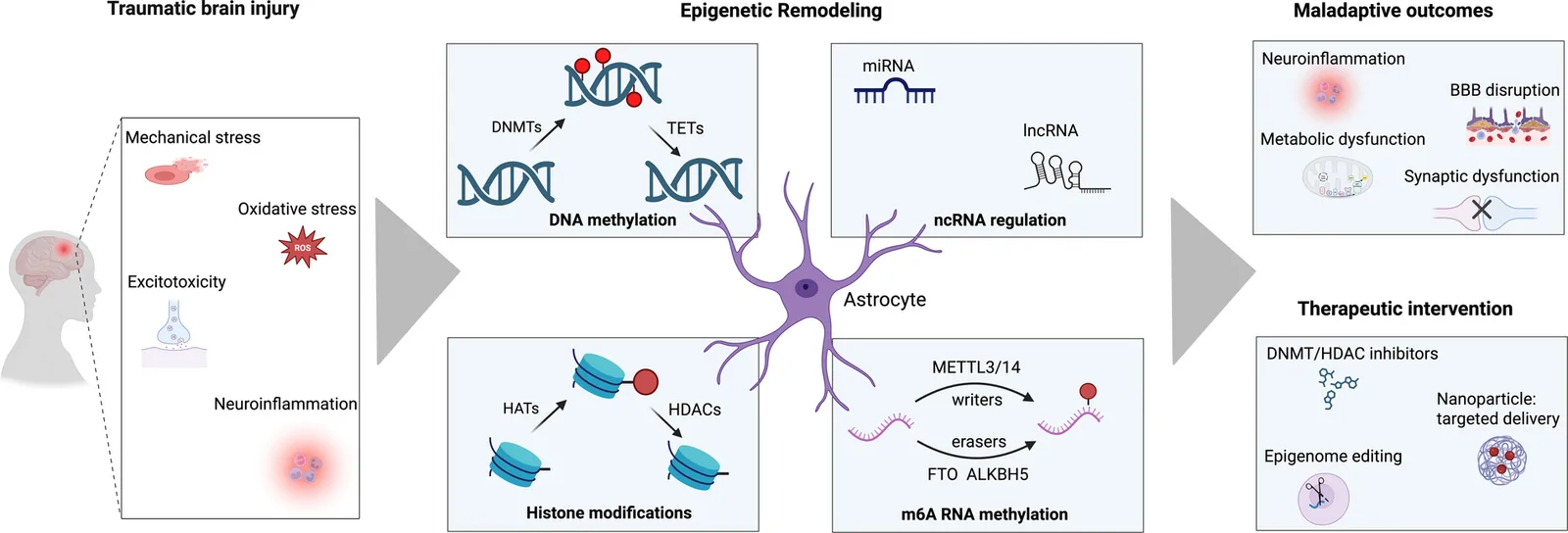
Epigenetic mechanisms in traumatic brain injury and their astrocyte-centered consequences. Traumatic brain injury (TBI) triggers multiple converging cellular stressors, including mechanical stress, oxidative stress, excitotoxicity, and neuroinflammation. Collectively they induce epigenetic remodeling in astrocytes. Four major epigenetic regulatory mechanisms are depicted: DNA methylation, mediated by DNA methyltransferases (DNMTs) and reversed by ten-eleven translocation (TET) enzymes; histone modifications, governed by the opposing activities of histone acetyltransferases (HATs) and histone deacetylases (HDACs); non-coding RNA regulation, including microRNAs (miRNA) and long non-coding RNAs (lncRNA); and N6-methyladenosine (m6A) RNA methylation, installed by writer complexes (METTL3/14) and removed by erasers (FTO, ALKBH5). The reactive astrocyte serves as the central integrator of these epigenetic signals. Persistent epigenetic dysregulation contributes to maladaptive outcomes including chronic neuroinflammation, blood–brain barrier (BBB) disruption, metabolic dysfunction, and synaptic dysfunction, collectively increasing the risk of neurodegeneration. Targeting these epigenetic mechanisms represents a promising therapeutic avenue, with strategies including DNMT/HDAC inhibitors, epigenome editing, and nanoparticle-based targeted delivery. Created with BioRender

TBI induces widespread and multi-layered epigenetic remodeling that reshapes transcriptional programs in the injured brain [10, 11, 19, 21]. These changes evolve dynamically over time. The primary epigenetic mechanisms are DNA methylation, histone modifications, and post-transcriptional regulation by ncRNAs (Fig. 1). These mechanisms act in concert to influence whether injury-induced transcriptional responses are transient or persist as long-term alterations in cellular state. Importantly, while these mechanisms are activated across multiple cell types, their persistence may be especially relevant in astrocytes, which integrate injury signals into long-term chromatin configurations. Representative epigenetic alterations reported across preclinical TBI models are summarized in Table 1.

**Table 1:** Summary of epigenetic changes in preclinical TBI studies

Epigenetic change	TBI model	Injury severity	Subject	Brain region	Cell type	Time points	Main Findings	Epigenetic modification and outcome	Reference
DNA Methylation	BI	mild	rat	frontal cortex	Neuron	8 mo	Distinct methylation profiles for neurons and glia, and an increase in global methylation in neuronal versus glial cells 8-month post TBI	N/R	[22]
	CCI	severe	mouse	hippocampus	N/R	4 mo	Profound changes in DNA methylation. Some differentially expressed genes are overlapping with demethylated CpG sites	Myo-inositol upregulate the expression of BATF2, a transcription factor involved in immune-regulatory networks	[23]
	CCI	N/R	mouse	perilesional cortex	Astrocyte	3 dpi	IRF1 + astrocytes are epigenetically regulated by TET3-mediated DNA hydroxymethylation and exacerbate TBI-associated pathology	IRF1 antagonist (8003–3282) alleviated inflammation and BBB damage, improved sensorimotor function and enhanced learning and memory abilities	[24]
	CCI	N/R	mouse	perilesional cortex	Astrocyte	N/R	NSUN2 enhances the stability of PTPRD mRNA through m5C methylation, which exacerbates the activation of the A1 astrocyte phenotype and intensifies the tissue damage caused by the inflammatory response	N/R	[25]
	FPI	N/R	rat	hippocampus	N/R	7 dpi	A large-scale switch of the methylation patterns	N/R	[26]
	SWI	N/R	mouse	perilesional cortex	Astrocyte	7 dpi	Highly reactive astrocytes exhibit 5-methylcytosine elevated	N/R	[16]
	WDI	N/R	rat	perilesional cortex	Astrocyte	4 dpi, 14 dpi	Nuclear as well as cytoplasmic Dnmt1 was observed in astrocytes	N/R	[27]
	WDI	mild	rat	hippocampus	N/R	7 dpi	Higher hippocampal protein levels of DNMT1 and DNMT3b	N/R	[28]
	WDI	Mild (repetitive)	rat	hippocampus	Neuron	30 dpi	Occupancy of DNMT3b at SOD2 promoter was higher at 30 dpi of the first episode of rMTBI and was sustained after the second episode of rMTBI	5-azacytidine, a pan DNMT inhibitor, normalized DNA methylation levels and revived SOD2 function after the second episode of rMTBI	[29]
	WDI	Mild (repetitive)	rat	hippocampus	N/R	30 dpi	DNA hypermethylation at the Mfn2 gene promoter	5-azacytidine normalized DNA methylation at Mfn2 promoter, which resulted in restoration of Mfn2 function	[30]
	WDI	Mild (repetitive)	rat	hippocampus	N/R	48 h, 30 dpi	Hypermethylation at TFAM promoter resulted in deficits in its protein levels. Hypomethylation of mitochondrial DNA (mtDNA) promoters (HSP1 and HSP2), which further culminated into low binding of TFAM	Methionine restored the methylation levels on HSP1 and HSP2 resulting in efficient binding of TFAM and normalized the rRNA, mRNA, and protein levels	[31]
	WDI	N/R	rat	perilesional cortex	Microglia/Macrophage	1 dpi, 2 dpi	Hypomethylation	Dexamethasone suppressed the lesional hypomethylation	[32]
Histone Modification	BI	N/R	rat	prefrontal cortex	Astrocyte	7 dpi	Decrease in H3 acetylation	N/R	[33]
	CCI	Mild (repetitive)	rat	hippocampus	N/R	48 h, 40 dpi	Impaired histone acetylation at CART promoter and CART expression	Trichostatin A (TSA), a pan HDAC inhibitor, restored deficits in recognition memory and HDAC activities	[34]
	CCI	N/R	mouse	spinal cord (post-TBI)	Neuron/Oligodendrocyte	35 dpi	HDAC2 mediates promoter deacetylation and chromatin compaction of BDNF genes, leading to a decrease in their expression	HDAC inhibitor (CI-994) administration in the chronic phase post-injury does not significantly affect the lesion volume and glial response and neuronal survival	[35]
	CCI	moderate	mouse	retina and optic nerve	RGC	7 dpi	Significant increase in the level of H3K9Me2 and G9a	G9a inhibitor, UNC0638 attenuates H3K9Me2 and activates Nrf2 to reduce oxidative stress, rescue the loss of retinal thickness, attenuate optic nerve degeneration and improved the retrograde transport of axons	[36]
	CCI	N/R	mouse	perilesional cortex	N/R	7 dpi	Increase acetylation of histone H3 at Lys14 and Lys27	Valproic acid (VPA) reduces hippocampal damage, microglial activation and pro-inflammatory gene expression	[37]
	FPI	N/R	rat	perilesional cortex	N/R	7 dpi	Protein levels of HDAC1 and HDAC3 were significantly elevated	Vorinostat (HDAC inhibitor) inhibited HDAC1, HDAC3, and Nox4 while activated AMPK signaling in ipsilateral injury cortical tissue	[38]
	WDI	Mild (repetitive)	rat	amygdala	N/R	48 h, 30 dpi	Dysregulation of BDNF expression due to dynamic interaction between histone deacetylase and DNA methylation modification at the BDNF gene promoter	Naïve Faecal microbiota transplantation, Lactobacillus rhamnosus GG administration, or Sodium butyrate treatment normalized chromatin state and BDNF levels, and reduced anxiety	[39]
	WDI	mild	rat	Multiple brain regions	Neuron	1–3 dpi, 7–8 dpi	HDAC4 and HDAC5 downregulated	Reduced levels of HDAC4 and HDAC5 expression observed in neurons were associated with the reduced nuclear and neuropil levels of HDAC4 and HDAC5	[40]
	WDI	N/R	rat	frontal cortex and subcortical	Neuron	7 dpi	p-H3S10 upregulated	U0126 (MEK1/2 inhibitor) reduced H3S10 phosphorylation and improved motor function recovery and cognitive function in both male and female rats	[41]
	WDI	severe	rat	perilesional cortex	Neuron	72 h	BET Family Protein BRD4 expression increased	N/R	[42]
RNA-Based Regulation	CCI	moderate	mouse	hippocampus	Neuron	6 h	Downregulation of METTL3 and m6A	N/R	[43]
	CCI	N/R	mouse	cerebral cortex	Neuron	6 h, 48 h	WTAP and m6A upregulation	N/R	[44]
	CCI	N/R	mouse	cortex	Microglia/Macrophage	1,3,7,14 dpi	Strong correlation between m6A modifications and genes enriched in microglia/macrophages across multiple time points after TBI. The m6A demethylase ALKBH5 was identified as a key regulator of dynamic m 6 A patterns at the injury site	N/R	[45]
	CCI	severe	rat	perilesional cortex	N/R	14 dpi	Decreased m6A modification and lower expression of METTL14, YTHDC2, and YTHDF1	The traditional Chinese herbal formula Xuefu Zhuyu decoction (XFZYD) ascends the expression of METTL14 and upregulates the m6A methylation level and the protein level of BDNF	[46]
	CCI	moderate/severe	rat	cortex	N/R	14 dpi	Dysregulation in miRNAs (miR-29b, miR-107b, miR-34, miR-181 and miR-9)	N/R	[47]
	CCI	N/R	mouse	cortex	Neuron/Microglia/Astrocyte	7 dpi	METTL3 downregulation	N/R	[48]
	FPI	N/R	rat	perilesional cortex	Astrocyte	2 wk	Higher miR155 and miR142 expression	N/R	[49]

*Abbreviations: BI* blast injury, *CCI* controlled cortical impact, *FPI* Fluid percussion injury, *SWI* stab wound injury, *WDI* weight drop injury, *RGC* retinal ganglion cell, *N/R* Not reported, *dpi* days post-injury

### DNA methylation

DNA methylation involves the addition of a methyl group to the C-5 carbon of cytosine bases to form 5’methyl-cytosine. This reaction is catalyzed by DNA methyltransferases (DNMTs) (Fig. 1). DNMT1 maintains existing methylation patterns, while DNMT3A and DNMT3B mediate de novo methylation at previously unmethylated loci [50]. Methylation at promoters and enhancers generally represses transcription by limiting transcription factor binding or recruiting repressive complexes. Active demethylation is mediated by ten-eleven translocation (TET) family enzymes (TET1, TET2, and TET3), which oxidize 5-methylcytosine through a stepwise process to facilitate its removal [50]. The dynamic interplay between DNMT and TET activity determines whether injury-responsive gene networks are silenced or accessible.

In rodent TBI models, hippocampal DNA methylation occurs within weeks [21], accompanied by upregulated DNMT expression [16, 27–29]. Genome-wide analyses reveal thousands of differentially methylated loci in both brain tissue and peripheral blood cells, with partial overlap between central and peripheral compartments [26]. An additional factor that may influence epigenetic signatures after TBI is the presence of intracranial hemorrhage. Blood-derived factors, iron accumulation, oxidative stress, and inflammatory mediators associated with hemorrhagic injury have the potential to alter gene regulatory programs and contribute to secondary brain injury [51]. Consistent with this concept, studies of intracerebral hemorrhage have implicated DNA methylation, histone modifications, and non-coding RNAs in injury progression and recovery [52]. Whether hemorrhagic and non-hemorrhagic forms of TBI generate distinct epigenetic signatures in astrocytes and other brain cell types remains largely unknown and warrants further investigation. Critically, many of these changes are region-specific and persist over time, a pattern consistent with stable epigenetic reprogramming rather than transient injury signaling [23].

The functional consequences of these methylation changes are best understood at specific loci. A recurring theme in TBI models is hypermethylation-driven silencing of homeostatic and neuroprotective genes [16]. DNMT3B-mediated hypermethylation of Sod2 in the hippocampus suppresses antioxidant defense and exacerbates oxidative stress and neuronal degeneration. DNMT inhibition restores Sod2 expression and improves learning and memory function [29]. Methylation of Mfn2 impairs mitochondrial function and contributes to bioenergetic failure, an effect reversed by DNMT inhibition [30]. Coordinated hypermethylation of nuclear TFAM and hypomethylation of mitochondrial DNA promoters disrupts mitochondrial biogenesis and reduces ATP production in the hippocampus. Methionine supplementation restores methylation patterns and normalizes mitochondrial function [31]. Hypermethylation of Aanat suppresses melatonin synthesis and has been associated with persistent sleep disturbances [22]. Given the strong associations between chronic sleep disruption, cognitive impairment, depression, anxiety, and neurodegenerative disease [53], epigenetic regulation of circadian and sleep-related pathways may represent an additional mechanism linking acute TBI to long-term neuropsychiatric dysfunction. Together, these findings reveal a consistent pattern: TBI biases the methylation landscape toward silencing of metabolic and homeostatic programs. This pattern is especially relevant to astrocytes, which are the principal regulators of brain metabolic homeostasis and in which injury-induced hypermethylation at homeostatic gene loci has been directly documented in the TBI penumbra [16].

In contrast, hypomethylation has been observed at inflammatory loci, particularly in perilesional microglia [32], which may promote sustained expression of inflammatory genes. This bidirectional methylation shift—silencing of protective programs alongside activation of inflammatory ones—creates a chromatin environment that amplifies secondary injury and may be particularly difficult to reverse once established.

Human studies corroborate the preclinical picture. Widespread alterations in DNA methylation of circulating leukocytes [54] and brain tissue [55] have been observed following TBI. Differentially methylated sites are enriched in genes involved in coagulation and inflammatory pathways [54]. In mild TBI, higher blood DNA methylation is associated with poorer episodic memory and severe anxiety [56]. In severe TBI, high DNA methylation of BDNF measured in genomic DNA extracted from the cerebrospinal fluid (CSF) was associated with better outcomes, suggesting its potential as an early biomarker for recovery [57]. In postmortem brain tissue of severe TBI, differentially methylated CpG sites are identified across neurodegeneration-related genes, with APP being the most affected [55]. This raises the possibility that TBI-induced methylation changes at these loci may mechanistically contribute to the elevated neurodegenerative risk observed epidemiologically.

### Histone modifications

Post-translational modifications of histone proteins regulate chromatin accessibility and transcriptional output by altering the physical compaction of chromatin and creating binding platforms for regulatory complexes. The most therapeutically studied modifications in TBI are acetylation and methylation, though phosphorylation and ubiquitination have also been documented following injury. Histone acetyltransferases (HATs) deposit acetyl groups at lysine residues, promoting open chromatin and transcriptional activation, while histone deacetylases (HDACs) reverse this mark and generally favor repression (Fig. 1). Histone methylation is catalyzed by enzymes such as euchromatic histone lysine methyltransferases (EHMTs). Histone methylation is more context-dependent: marks such as H3K4me1 are associated with enhancer activation, while H3K9me2 and H3K27me3 are associated with transcriptional repression and heterochromatin formation. TBI disrupts the balance across all these modifications, with consequences differing by cell type, brain region, and injury phase.

The most consistent finding across TBI models is a shift toward repressive chromatin states at neuroprotective and homeostatic loci [58]. Increased expression of HDAC2-5 and HDAC11 has been reported in the cortex, hippocampus, and amygdala regions following injury [34, 39], while HDAC4-5 show region- and time-dependent downregulation [40]. HDAC2-mediated repression reduces the expression of synaptic plasticity-related genes including BDNF and synaptophysin [59]. HDAC inhibition or HDAC2 knockdown enhances BDNF expression and promotes synapse formation and function recovery after brain injury in rodent models [35]. In astrocytes specifically, reduced acetylation of histones H2B, H3, and H4 has been documented following blast injury and correlates with increased reactivity [33], suggesting that the global shift toward deacetylation is not confined to neurons but shapes astrocyte chromatin states as well.

Histone methylation adds further regulatory complexity. Enhancer activation at H3K4me1-marked loci increases WNT3A expression, which promotes neurite extension and regrowth [60]. Conversely, EHMT2 (G9a)-mediated elevation of H3K9me2 restricts Nrf2 binding to antioxidant gene promoters and exacerbates oxidative stress [36]. This repressive mechanism parallels the DNMT-mediated silencing of Sod2 described above, and both converge on the same functional outcome of impaired antioxidant defense. Loss of the repressive H3K27me3 mark promotes cellular senescence and contributes to a chronic neuroinflammatory environment [61]. Notably, elevated H3K9me2 in tauopathy models correlates with Tau hyperphosphorylation while EHMT inhibition reduces pathological Tau burden [62], linking chromatin regulation directly to the neurodegenerative sequelae that TBI epidemiology predicts.

Histone phosphorylation provides an additional layer of injury-responsive regulation. Phosphorylation of histone H3 at serine 10 (p-H3S10) is significantly elevated in the brain in both rodent TBI models and human patients [41]. This sustained upregulation is driven by persistent activation of upstream ERK1/2, which directly phosphorylate H3S10. Pharmacologic inhibition of ERK reduces p-H3S10 levels, attenuates neuroinflammation, and improves neurological outcomes [41]. These findings suggest that ERK-driven H3S10 phosphorylation contributes to TBI-associated neuroinflammatory pathology.

Across modification types, a coherent picture emerges: TBI shifts the histone landscape toward states that silence homeostatic and neuroprotective programs while permitting or amplifying inflammatory gene expression. This pattern closely mirrors TBI-induced DNA methylation changes and reinforces the view that multiple epigenetic mechanisms converge on a shared maladaptive transcriptional outcome. In astrocytes, where histone acetylation directly governs reactivity state transitions and the expression of hallmark markers such as GFAP and VIM [63], this convergence may be especially consequential for the persistence of injury-induced chromatin configurations.

### RNA-based regulation

Beyond chromatin-level modifications, TBI alters RNA-based regulatory systems that control gene expression post-transcriptionally. These mechanisms can interact with and reinforce chromatin-level changes, adding a further dimension to the injury-induced regulatory landscape.

N6-methyladenosine (m6A) is the most prevalent post-transcriptional epigenetic modification in the mammalian CNS, regulating mRNA stability, splicing, and translation [64]. Although m6A operates at the epitranscriptomic rather than chromatin level, it is increasingly discussed alongside classical epigenetic mechanisms because of its broad influence on gene expression programs [65]. m6A deposition is mediated by writer complexes composed of a catalytic METTL3 (Methyltransferase-like 3) and a structurally-important METTL14 (methyltransferase-like 14), and is reversed by demethylases including FTO and ALKBH5 [66] (Fig. 1). TBI induces widespread changes in m6A RNA methylation, affecting transcripts involved in metabolism, development, and inflammation [43]. The functional consequences are bidirectional and context-dependent. WTAP-dependent m6A modification of NLRP3 mRNA recruits the reader YTHDF1 to enhance inflammasome translation and exacerbates neuronal damage [44]. In contrast, ALKBH5-mediated m6A in the SOCS3 mRNA in microglia/macrophages stabilizes SOCS3 protein to suppress neuroinflammation [45]. METTL14-mediated regulation of Bdnf mRNA further affects neurotrophic signaling during recovery [46]. This bidirectionality mirrors the opposing methylation and acetylation patterns described above: a recurring theme in TBI epigenomics where the same class of regulatory mechanism can drive either injury amplification or repair depending on the cellular and molecular context.

Non-coding RNAs represent an additional layer of epigenetic regulation in the TBI response. MicroRNAs (miRNA) such as miR-29b [47] and long non-coding RNAs (lncRNA) (Fig. 1) including 4933431K23Rik [67] have been implicated in chronic neuroinflammation, Tau phosphorylation, and synaptic dysfunction following injury. These molecules are highlighted as representative examples of the broader spectrum of non-coding RNAs altered after TBI. A recent systematic review identified 37 differentially expressed miRNAs after TBI, 10 of which were computationally predicted by Ingenuity Pathway Analysis to regulate 15 genes associated with human neurological disease [10]. This highlights the breadth of miRNA involvement, though the functional relevance of most individual miRNAs in specific cell types remains to be established. The cell-type-enriched expression of many non-coding RNAs, including astrocyte-enriched species, raises the possibility that RNA-based mechanisms may contribute to astrocyte-specific transcriptional reprogramming after TBI, complementing the chromatin-level changes described in sections "DNA methylation" and "Histone modifications".

### Temporal dynamics of epigenetic changes after TBI

TBI progression unfolds across acute, subacute, and chronic phases, each associated with distinct pathological processes [3]. Epigenetic mechanisms track this progression closely, dynamically reshaping gene expression programs that govern the intensity of secondary injury and the trajectory of functional recovery [11, 68]. Understanding the temporal profile of epigenetic changes is essential for interpreting which modifications reflect transient injury responses and which represent stable reprogramming with long-term functional consequences.

In the acute phase, epigenetic modifications are rapid and reversible in principle [69], yet they can still drive significant neuronal damage [44], oxidative stress [29], mitochondrial dysfunction, and ferroptosis [48] before resolution occurs. The speed of these changes suggests that rather than by transcriptional adaptation, they are instead driven by immediate injury signals, such as mechanical stress, excitotoxic calcium influx, and reactive oxygen species. They may establish an early chromatin environment that constrains subsequent regulatory possibilities.

As injury progresses into the chronic phase, a subset of epigenetic changes stabilizes rather than resolves. Persistent enhancer activation, sustained DNA hypermethylation at homeostatic gene loci, and prolonged changes in histone modification profiles have all been documented in the weeks to months following injury [56, 70, 71]. Crucially, not all cell types contribute equally to this persistence. Neurons, which are post-mitotic and subject to ongoing death in the perilesional zone, may lose epigenetic marks simply through cell loss. Astrocytes, by contrast, are long-lived, proliferate modestly after injury, and maintain chromatin states across cell divisions, making them particularly likely candidates for carrying persistent epigenetic modifications forward in time [72].

The stage-specificity of these effects is further illustrated by evidence that epigenetic perturbations during early but not late postnatal astrocyte development produce lasting chromatin remodeling and inflammatory predisposition, suggesting that discrete temporal windows govern the durability of astrocyte epigenetic reprogramming [73].

This temporal pattern has direct implications for therapeutic targeting. Interventions timed to the acute phase may reverse early modifications before they stabilize, but risk interfering with protective epigenetic responses that also operate in this window. Chronic-phase interventions face a more consolidated chromatin landscape that may be less responsive to pharmacologic modulation. The phase-dependence of epigenetic changes therefore argues against a single therapeutic window and in favor of stage-matched intervention strategies, a point discussed further in section "Therapeutic targeting of astrocyte epigenetic states".

The persistence of epigenetic modifications across the chronic phase suggests that TBI may leave a lasting molecular imprint on the brain. This persistence provides the conceptual foundation for the astrocyte epigenetic memory hypothesis introduced in the following sections: the idea that injury-induced chromatin configurations, once stabilized, can shape astrocyte behavior and inflammatory responsiveness long after the initiating insult has resolved.

An important but understudied consideration is whether epigenetic remodeling differs across TBI models and injury severities. Focal injury models such as controlled cortical impact (CCI) and fluid percussion injury (FPI) produce substantial tissue damage, reactive gliosis, and BBB disruption, whereas diffuse and repetitive mild TBI models are typically characterized by less overt structural pathology but persistent network dysfunction and chronic neuroinflammatory responses [74, 75]. These distinct biomechanical insults likely engage different mechanotransduction, inflammatory, and metabolic pathways, which may in turn influence chromatin remodeling trajectories [76]. Consequently, the initiation, persistence, and functional consequences of astrocytic epigenetic reprogramming may vary according to injury type, severity, and post-injury interval. Systematic comparative studies across TBI models remain limited and represent an important area for future investigation. Consequently, the initiation, persistence, and functional consequences of astrocytic epigenetic reprogramming may vary according to injury type, severity, and post-injury interval, as illustrated by the diversity of preclinical studies summarized in Table 1.

## Astrocyte-specific epigenetic regulation in TBI

Astrocytes are the most abundant glial cells in the central nervous system (CNS). They perform essential functions in maintaining CNS homeostasis, including regulation of synaptic and circuit activity, cerebral blood flow, and metabolic support to neurons [15, 77]. Their functional identity is highly plastic and is shaped in part by epigenetic mechanisms [18], enabling adaptive responses to developmental and environmental cues.

In pathological contexts, astrocytes undergo context-dependent transitions into reactive states characterized by pronounced changes in morphology, metabolism, cytokine and chemokine production, and dynamic interactions with other cells in the brain [14]. Epigenetic regulation plays a central role in governing these state transitions and in shaping astrocyte reactivity and phenotype [18].

Following TBI, astrocytes adopt reactive reprograms that evolve across injury phases and may persist beyond the acute period. In a mouse CCI model, astrocyte response shifts from an acute, predominantly pro-inflammatory profile (marked by upregulated Gfap, Tnf, and interferon/TNF-α signaling), through a subacute mixed inflammatory-neurodegenerative state (including Sparc, C1qa, and Trem2), to a chronic profile resembling neurodegenerative signatures (marked by upregulated Apoe, Mapt, App and App accumulation) [78]. These phase-dependent dynamic shifts suggest that astrocyte identity is continuously remodeled after TBI, a process likely driven, at least in part, by the epigenetic mechanisms discussed in the preceding section.

### Epigenetic regulation of astrocyte state transitions

Reactive astrocytes exhibit substantial heterogeneity, with distinct molecular programs and signaling pathways governing state-specific phenotypes [79]. Key regulators include JAK-STAT3, TGF-β-Smad, NF-κB, Olig2, and NFAT [80]. Epigenetic mechanisms integrate these signals to shape transcriptional output and state stability of astrocyte reactivity [18].

DNA methylation also shapes astrocyte state regulation. MAFG cooperates with MAT2α to drive methylation-dependent repression of NRF2-responsive antioxidant genes in MS and EAE models [81], while histone acetylation at inflammatory loci — supported by ACLY-derived acetyl-CoA and p300 — enhances pro-inflammatory transcription [82]. These opposing modifications underscore the bidirectional nature of epigenetic control in astrocyte activation, which extends even to structural hallmarks of reactivity: GFAP and VIM expression is itself regulated at the chromatin level [83]. Further evidence comes from an Alzheimer's disease model, where astrocyte-specific Mettl14 knockout on the APP/PS1 background reduced astrogliosis, attenuated neuroinflammation, and improved cognition [84]. Mechanistically, Mettl14-mediated m6A modification promoted astrocyte inflammatory signaling through MAPK pathway activation, highlighting RNA modifications as an additional layer of astrocyte state regulation. Pharmacologic studies further highlight the significance of epigenetic mechanisms in astrocyte reactivity. In a phenotypic screening platform, HDAC3 inhibitors emerged as potent suppressors of pathological reactive states, attenuating pro-inflammatory and neurotoxic gene expression while promoting neuroprotective signatures in astrocytes [85]. These findings reinforce the importance of epigenetic mechanisms in regulating astrocyte reactivity and point toward the therapeutic potential of HDAC inhibition for dampening chronic neuroinflammation. Collectively, these findings demonstrate that astrocyte phenotypes are dynamically regulated by diverse epigenetic mechanisms across CNS disorders. This growing body of evidence provides a working model for understanding how epigenetic remodeling may govern astrocyte state transitions following TBI.

### Epigenetic remodeling in reactive astrocytes after TBI

Reactive astrocytes in TBI undergo substantial epigenetic remodeling that reshapes transcriptional programs governing inflammation, metabolism, stress responses, and extracellular matrix organization. TBI induces significant DNMT1 re-localization in reactive astrocytes, shifting from the strictly neuronal nuclear localization in sham animals to a dual nuclear and cytoplasmic distribution in animals with TBI [27]. This change suggests injury-associated disruption of methylation machinery. Acetylation of histones H2B, H3, and H4 is reduced in astrocytes following blast injury, correlating with increased astrocyte reactivity [33]. In the TBI penumbra, highly reactive astrocytes exhibit persistent DNA hypermethylation, accompanied by decreased expression of homeostatic genes [16]. This hypermethylated state is associated with upregulation of MAFG-1, DNMT1, and DNMT3a, suggesting activation of methylation-dependent repressive machinery. Together, these findings support the presence of sustained chromatin remodeling in reactive astrocytes after TBI, characterized by reduced histone acetylation and enhanced DNA methylation at loci linked to homeostatic gene regulation.

The functional significance of epigenetic remodeling in astrocytes is illustrated by recent mechanistic work on DNA hydroxymethylation. TET3-mediated conversion of 5-methylcytosine to 5-hydroxymethylcytosine has been identified as an epigenetic mechanism upregulating IRF1 expression in astrocytes after TBI [24]. Elevated IRF1 binds to promoters of inflammatory cytokine genes and activates NF-κB- and STAT-dependent transcriptional programs. This triggers an inflammatory cascade that ultimately drives BBB breakdown and cerebral edema. Importantly, astrocyte-specific Irf1 deletion or pharmacologic inhibition of IRF1 attenuates inflammatory signaling and neurovascular pathology [24]. These findings provide mechanistic evidence that TBI-induced epigenetic modifications in astrocytes can drive sustained neuroinflammation and contribute to secondary injury progression.

Although this review focuses on astrocytes, their epigenetic changes are not isolated events. Following TBI, microglia are among the earliest responders to injury and are major sources of inflammatory mediators that shape astrocyte activation [86]. Cytokines and complement factors released by activated microglia, including IL-1α, TNF, and C1q, promote the transition of astrocytes toward reactive phenotypes [87] and influence their transcriptional programs. Importantly, the concept of epigenetically encoded innate immune memory is now well established in microglia and other innate immune cells [88]. Experimental studies have demonstrated that prior inflammatory or pathological stimuli can induce persistent epigenetic alterations that subsequently modify microglial responses to secondary challenges [89]. These findings provide proof-of-principle that long-lasting, epigenetically regulated memory states can occur in resident glial populations. Recent multi-omic studies in TBI have demonstrated that aging-associated shifts toward glycolysis in microglia are accompanied by widespread chromatin accessibility changes at pro-inflammatory regulatory elements [90]. These findings suggest that persistent glial phenotypes may emerge through coordinated metabolic-epigenetic reprogramming across multiple cell populations. Astrocytes are uniquely positioned to influence multiple aspects of chronic TBI pathology through their roles in metabolic support, neurotransmitter clearance, ion and water homeostasis, BBB maintenance, and neurovascular coupling. Consequently, persistent astrocytic epigenetic alterations may have particularly broad functional consequences following injury. Understanding how microglial and astrocytic epigenetic programs interact will therefore be essential for defining the mechanisms underlying chronic neuroinflammation and putative astrocytic epigenetic memory after TBI.

### Toward an astrocytic epigenetic memory hypothesis

Epigenetic memory refers broadly to the persistence of chromatin states or epigenetic marks following an initial stimulus, resulting in altered cellular responses to subsequent challenges. A well-established example of this phenomenon is trained immunity, a form of innate immune memory in which prior stimulation induces long-lasting functional reprogramming through coordinated epigenetic and metabolic remodeling [91, 92]. In innate immune cells, including microglia, such memory-like states are characterized by persistent changes in chromatin accessibility, histone modifications, and cellular metabolism that shape responses to later stimuli [91, 92]. Whether analogous memory-like states occur in astrocytes following TBI remains unknown. Nevertheless, the possibility that injury-induced chromatin remodeling may persist beyond the acute phase and influence astrocyte responses to subsequent inflammatory or injury-related challenges provides the conceptual basis for the astrocytic epigenetic memory hypothesis discussed below.

The observation that TBI-induced chromatin remodeling can sustain inflammatory transcription raises an important question: are these epigenetic alterations transient consequences of acute injury, or can they persist as stable molecular imprints that shape future astrocyte behavior?

Emerging evidence suggests that at least a subset of these changes may be durable. Persistent DNA hypermethylation, sustained enhancer activation at injury-responsive loci, and prolonged accessibility of subsets of injury-responsive enhancers (IRENs) have been documented in astrocytes following CNS injury [16, 93]. Notably, studies in spinal cord injury have demonstrated that astrocyte-specific IRENs integrate stress-responsive transcription factor motifs such as AP-1 with lineage-defining factors including SOX9 and NFIA [93]. Although these findings were derived from spinal cord injury rather than TBI, they provide important proof-of-principle that CNS trauma can induce durable enhancer remodeling in astrocytes. While most injury-induced transcriptional changes resolve over time, a subset of early-commissioned IRENs remains accessible up to 28 days post-injury. Notably, 665 of 1,027 astrocyte injury-induced genes are associated with these newly established enhancers, suggesting that astrocyte reactivity is primarily orchestrated by the recruitment of IRENs [93]. This prolonged enhancer accessibility is consistent with the concept of epigenetic memory and support that early injury signals may establish durable chromatin configurations in astrocytes.

Evidence from other neuroinflammatory contexts suggests that astrocytes are capable of acquiring memory-like states. In models of multiple sclerosis and experimental autoimmune encephalomyelitis (EAE), subsets of ACLY⁺ p300⁺ astrocytes retain sustained histone acetylation and open chromatin at proinflammatory enhancers after resolution of the initiating stimulus [82]. This persistent accessibility enables accelerated or amplified transcriptional responses upon rechallenge, resembling trained immunity in myeloid cells [72]. Importantly, this memory-like state reflects durable chromatin remodeling rather than continued exposure to inflammatory cues.

Additional support comes from a recent study demonstrating that the loss of NR3C1 (encoding the glucocorticoid receptor), which functions as a key regulator of early postnatal astrocyte development, epigenetically predisposes inflammatory gene programs [73, 94]. Although NR3C1-deficient astrocytes show no phenotypic abnormalities at baseline, they mount exacerbated immune responses upon EAE induction in adulthood, driven by persistently altered chromatin accessibility at inflammatory loci established during early development [73].

Further insight into the relationship between injury and astrocyte epigenetic remodeling comes from a study demonstrating that ischaemic injury reprograms the astrocyte DNA methylome toward a stem cell-like state in a DNMT3A-dependent manner [95]. Notably, these ischaemia-induced methylome changes resolved within 21 days post-injury, indicating that not all injury-induced epigenetic remodeling is persistent. This stands in contrast to the persistent histone acetylation and open chromatin at proinflammatory enhancers observed in EAE models, where chromatin remodeling is retained after resolution of the initiating stimulus [82]. It should be noted, however, that Kremer et al. assessed injury-induced methylome remodeling specifically in subventricular zone and striatal astrocytes undergoing neurogenic reprogramming [95]. Because the study focused on neurogenic astrocytes rather than reactive cortical astrocytes, whether similar dynamics occur in TBI-relevant astrocyte populations remains unknown.

In addition, findings from human iPSC-derived astrocytes support the concept of durable priming [96]. Human iPSC-derived astrocytes can retain altered responsiveness after stimulus withdrawal and exhibit amplified responses to secondary Aβ exposure, consistent with durable priming.

Mechanistic studies further reinforce this model. TET3-mediated DNA hydroxymethylation promotes sustained IRF1 expression in astrocytes after TBI, driving proinflammatory transcriptional programs and neurovascular pathology [24]. This study provides a DNA modification-based mechanism for prolonged inflammatory gene reinforcement. Although formal rechallenge experiments have not yet been performed in TBI models to directly demonstrate priming, the combination of persistent enhancer accessibility, stable DNA modification changes, and prolonged reactive gene expression are consistent with the emergence of a memory-like astrocyte state. Collectively, current TBI studies satisfy several proposed features of epigenetic memory, including injury-induced chromatin remodeling, persistence beyond the acute injury phase, and sustained effects on gene expression. However, evidence demonstrating altered responses to a secondary challenge—a defining feature of memory-like states—remains lacking.

**Fig. 2:**
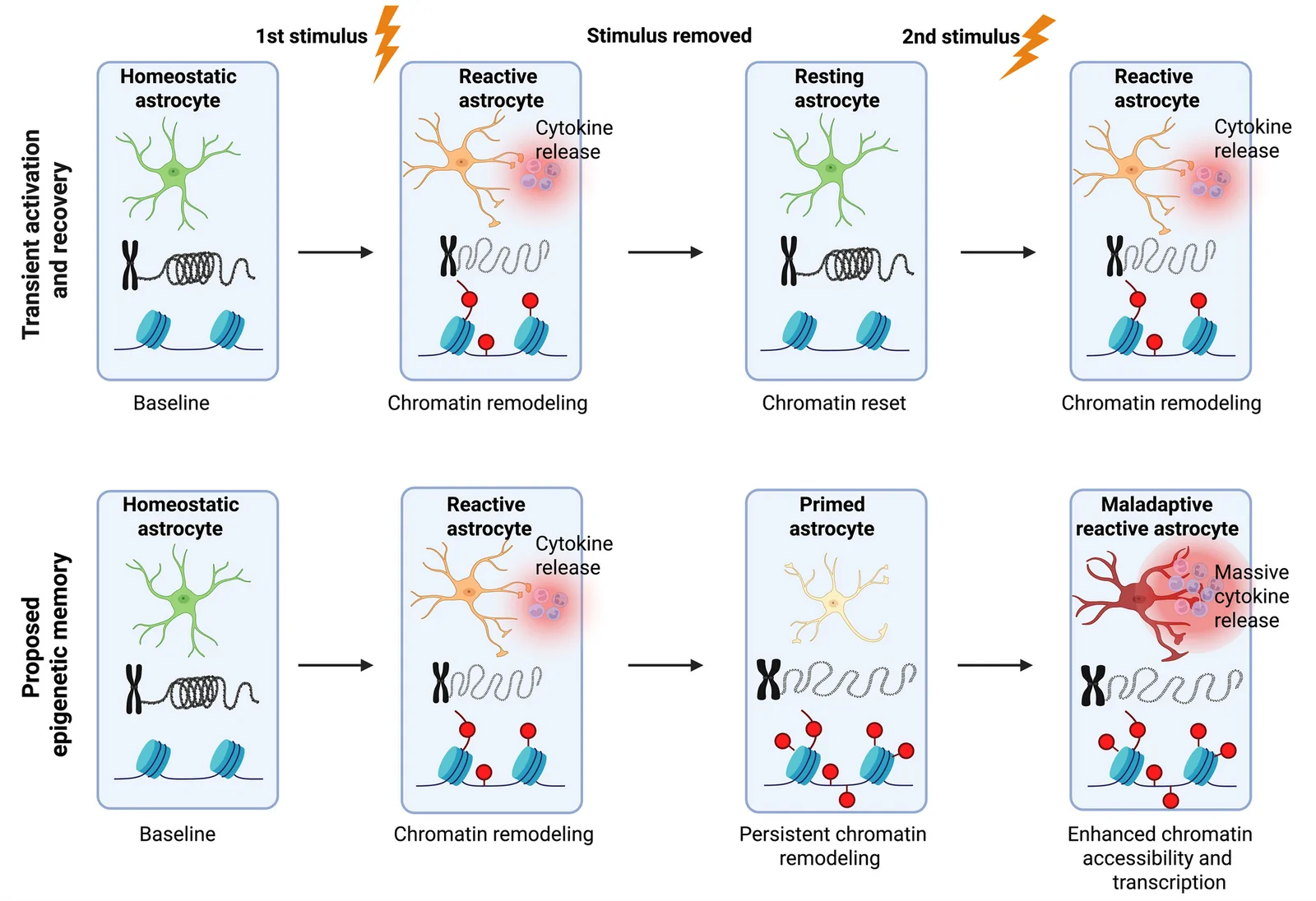
Conceptual framework for transient astrocyte activation versus proposed astrocytic epigenetic memory. Upper panel – Transient activation and recovery. An initial inflammatory or injury-related stimulus induces astrocyte reactivity, accompanied by chromatin remodeling, cytokine production, and morphological changes. Following stimulus removal, the chromatin landscape returns to baseline and the astrocyte resumes a resting state. Upon a subsequent challenge, the astrocyte mounts a response comparable in magnitude and duration to the initial one, indicating no lasting functional imprint from prior exposure. Lower panel – Proposed astrocytic epigenetic memory. The initial stimulus similarly induces chromatin remodeling, cytokine production, and reactive morphological changes. However, after stimulus withdrawal, a subset of injury-induced chromatin alterations persists, leaving the astrocyte in a primed or memory-like state. Upon a subsequent challenge, the astrocyte exhibits an altered response, potentially enhanced, accelerated, prolonged, or otherwise modified by prior exposure. While astrocytic epigenetic memory has not been definitively demonstrated after TBI, this model illustrates a proposed mechanism by which persistent chromatin remodeling could shape future astrocyte behavior. Red circles represent activating epigenetic marks (e.g., histone acetylation such as H3K27ac), and chromatin schematics illustrate shifts from relatively closed to more accessible configurations associated with increased transcriptional potential. Created with BioRender

Within this model, acute mechanical injury may induce lasting shifts in astrocytic regulatory architecture that bias subsequent responses to secondary challenges (Fig. 2). Determining whether such chromatin configurations represent true epigenetic memory will require longitudinal and rechallenge-based experimental designs. The growing body of evidence from EAE, ischemia, and developmental models collectively suggests that astrocytes are capable of encoding prior experiences in their chromatin. Additionally, the durability and functional consequences of this encoding depend on the nature of the stimulus, the developmental stage of the cell, and the regional identity of the astrocyte population. Applied to TBI, where perilesional astrocytes are exposed to sustained, multifactorial pathological signals, this framework support the hypothesis that acute injury can imprint durable regulatory states, potentially linking early trauma to long-term neuroinflammatory and neurodegenerative vulnerability.

## Epigenetics as a link between astrocyte reactivity and TBI pathophysiology

Astrocytes are central regulators of neuroinflammation, metabolic homeostasis, synaptic integrity, and vascular stability [97–99]. Following TBI, epigenetic remodeling reshapes astrocytic transcriptional programs in ways that extend beyond acute reactivity, influencing long-term tissue architecture and functional recovery (Fig. 1). Rather than acting as passive responders, reactive astrocytes actively shape the trajectory of secondary injury.

### Neuroinflammation

Reactive astrocytes contribute to sustained inflammatory signaling through cytokine release, chemokine production, and amplification of microglial activation [7–9]. Epigenetic modifications, including enhancer acetylation and altered DNA methylation at inflammatory loci, stabilize transcriptional programs governing NF-κB, JAK-STAT, and interferon pathways. A notable example is TET3-mediated hydroxymethylation, which promotes IRF1 expression in reactive astrocytes and drives production of pro-inflammatory mediators including IL-6 and TNF-α. These changes are associated with increased complement activation, recruitment of peripheral immune cells, and blood–brain barrier dysfunction.

Persistent chromatin accessibility at pro-inflammatory genes may sustain reactive astrocyte states beyond the initial insult, reinforcing microglial activation and maintaining a self-perpetuating inflammatory microenvironment. Thus, epigenetic remodeling may convert transient inflammatory responses into chronic neuroinflammation, a process increasingly implicated in long-term neurological dysfunction and neurodegeneration after TBI. However, although epigenetic changes are associated with persistent neuroinflammation following TBI, whether the persistence of these epigenetic alterations directly drives or maintains chronic neuroinflammation remains elusive [100].

### Metabolic dysregulation

Astrocytes support neuronal metabolism through lactate shuttling, mitochondrial regulation, and antioxidant defense [15, 77]. TBI-associated epigenetic changes at metabolic and mitochondrial genes impair these functions [16]. For example, DNMT3A-mediated hypermethylation of LDHA and Slc16a1 suppresses genes required for lactate production and transport, disrupting the astrocyte-neuron lactate shuttle and reducing metabolic support to injured neurons. In parallel, hypermethylation of genes such as Mfn2 and TFAM, together with repressive histone marks at antioxidant loci, impairs mitochondrial integrity and redox balance [30, 31]. These alterations compromise astrocytic metabolic support, increase oxidative stress, compromise neuronal resilience, and exacerbate secondary injury. Because astrocytes occupy a central position in brain energy metabolism, persistent epigenetic dysregulation may connect inflammatory activation with metabolic insufficiency, thereby amplifying chronic neurological dysfunction after TBI.

### BBB disruption and vascular remodeling

Astrocytic endfeet ensheath cerebral vasculature and are essential for BBB integrity [15, 77]. Astrocyte endfeet stabilize the BBB through tight junction regulation, aquaporin-mediated water transport, and modulation of inflammatory signaling. Epigenetic dysregulation compromises this support through multiple mechanisms. Injury-induced hypermethylation in reactive astrocytes leads to decreased expression of the water channel aquaporin-4 (Aqp4) [16], impairing astrocytic water homeostasis. Reactive astrocytes with IRF1-mediated inflammatory programs release matrix metalloproteinases and cytokines that degrade extracellular matrix and disrupt tight junctions, thereby increasing BBB permeability [24]. The resulting influx of peripheral immune cells and blood-borne molecules into the brain parenchyma amplifies local inflammation. This creates a self-reinforcing cycle in which epigenetically reprogrammed reactive astrocytes fail to maintain vascular integrity, further worsening the injury environment [101, 102]. Epigenetic changes in perivascular astrocytes thus translate injury signals into neurovascular pathology with consequences in secondary injury and long-term tissue damage.

### Synaptic dysfunction and neurodegeneration

Astrocytes regulate synaptic transmission through glutamate uptake, ion buffering, and trophic support. Epigenetic repression of glutamate transporter genes and neurotrophic factors diminishes synaptic stability and plasticity [77, 103]. For example, downregulation of GLT-1/EAAT2 transporters and Kir4.1 channels following TBI can impair glutamate clearance and potassium buffering, promoting excitotoxicity. Epitranscriptomic modifications, such as NSUN2-mediated m5C methylation of PTPRD mRNA, further exacerbate neurotoxic astrocyte activation, with knockdown of NSUN2 mitigating neuronal death [25].

Persistent post-TBI epigenetic marks overlap with loci implicated in neurodegenerative disease, including amyloid precursor protein (APP), MAPT (encoding Tau protein isoforms), and neurofilament genes (NEFH, NEFM, and NEFL) [55]. This finding suggests that astrocytic chromatin reprogramming may lower the threshold for later pathological cascades and may play a role in post-TBI Aβ or Tau accumulation. Shared epigenetic signatures between TBI and neurodegenerative conditions indicate convergence on maladaptive regulatory states [100]. Epidemiological and experimental data support that such epigenetic modification contributes to cognitive decline, neuropsychiatric sequelae, and increased risk of neurodegenerative disease in susceptible individuals [104–106]. Dysregulated chromatin states reduce astrocyte-mediated glutamate recycling, impair neurotrophic factor secretion including BDNF and GDNF, and promote the release of neurotoxic factors associated with complement-activating reactive astrocyte phenotypes. Collectively they disrupt the synaptic support that astrocytes normally provide [16]. Treatment with a DNMT inhibitor restored expression of homeostatic genes and prevented the neurotoxic effects of reactive astrocytes. This positions persistent astrocyte epigenetic reprogramming as a mechanistic link between acute TBI and the increased risk of neurodegenerative diseases associated with prior brain injury.

TBI is increasingly recognized as a risk factor for multiple neurodegenerative disorders, most notably Alzheimer's disease, but also Parkinson's disease, amyotrophic lateral sclerosis, and related dementias [7]. However, the mechanisms linking an acute injury to neurodegeneration that emerges years or decades later remain incompletely understood. Epigenetic remodeling offers a potential molecular mechanism for this connection [19, 100]. By establishing persistent alterations in astrocyte inflammatory, metabolic, and neurovascular programs, injury-induced epigenetic changes may promote chronic neuroinflammation, impaired proteostasis, synaptic dysfunction, and reduced resilience to age-related pathology. Alzheimer's disease is currently the best-characterized example of this relationship, providing substantial evidence that chronic glial dysfunction and persistent inflammatory signaling contribute to neurodegenerative progression following TBI. However, the astrocyte-specific epigenetic mechanisms linking TBI to neurodegeneration remain incompletely understood and represent an important area for future investigation.

Beyond neurodegeneration, astrocyte epigenetic remodeling may also contribute to post-traumatic epileptogenesis, a major long-term complication of TBI [107]. Astrocytes play critical roles in maintaining network stability through glutamate uptake, potassium buffering, water homeostasis, and BBB support. Disruption of these functions has been implicated in seizure generation and epileptogenesis [108]. Reactive astrocytes also contribute to chronic neuroinflammation and BBB dysfunction, both of which are recognized drivers of epileptogenic network remodeling following TBI [107]. Recent studies further demonstrate that cortical astrocytes from animals that develop post-traumatic epilepsy exhibit distinct injury-associated transcriptional signatures [109, 110], supporting a role for astrocytic reprogramming in epileptogenesis. Persistent epigenetic repression of astrocyte homeostatic genes after TBI may therefore create a pro-epileptogenic microenvironment characterized by impaired glutamate clearance, extracellular potassium accumulation, chronic inflammation, and heightened neuronal excitability [111]. Future studies are needed to determine whether targeting astrocyte epigenetic states can mitigate epileptogenesis and improve long-term neurological outcomes after TBI.

Apolipoprotein E4 (APOE4) is the strongest genetic risk factor for the sporadic late-onset form of Alzheimer’s disease [112]. In TBI, the APOE4 allele similarly worsens outcomes: APOE4 carriers have higher odds of poor long-term recovery [113], and APOE4 presence is associated with poorer memory performance across all TBI severities in military personnel [114]. Astrocytes are the principal source of APOE in the CNS [112]. Astrocyte-produced APOE4 can promote BBB damage, impair tau clearance, and reduce neuron support and metabolism [112]. APOE expression in astrocytes is subject to epigenetic regulation [115]. In Alzheimer's disease brains with APOE4/4, European local ancestry at the APOE locus is associated with increased chromatin accessibility at the APOE promoter and elevated APOE4 expression in astrocytes [116]. These epigenetic differences are associated with genome-wide astrocytic accessibility differences enriched for genes involved in synaptic processes, cholesterol metabolism, and astrocyte reactivity. Together, these findings suggest that chromatin-level regulation of APOE in astrocytes may represent a point of convergence between genetic risk, acute injury, and long-term neurodegeneration.

### Epigenetic aging and astrocyte senescence

An emerging question is whether persistent astrocyte epigenetic remodeling following TBI represents a form of accelerated biological aging [117]. TBI is increasingly recognized as a risk factor for age-related neurodegenerative disorders, including Alzheimer's disease and related dementias [118], and several studies suggest that injury can induce molecular signatures resembling those observed during normal aging [119]. Aging is accompanied by characteristic epigenetic alterations, including changes in DNA methylation patterns, chromatin accessibility, histone modifications, and transcriptional regulation [120]. Similar processes occur following TBI, where persistent inflammatory signaling and metabolic dysfunction could stabilize maladaptive chromatin states in reactive astrocytes.

Recent studies have demonstrated that aging profoundly alters glial metabolic and epigenetic programs [121]. Integrated multi-omic analyses of aged TBI brains identified pro-inflammatory microglial states associated with glycolytic rewiring and widespread changes in chromatin accessibility, highlighting a close relationship between metabolic state and epigenetic regulation [90]. Whether analogous mechanisms operate in astrocytes remains unclear, but sustained enhancer remodeling, persistent inflammatory gene activation, and impaired metabolic support could promote a senescence-like phenotype characterized by chronic cytokine production and diminished homeostatic capacity. Such changes may contribute to the senescence-associated secretory phenotype (SASP), perpetuating neuroinflammation and accelerating neurodegenerative processes [121].

Importantly, the relationship between astrocytic epigenetic memory and cellular senescence remains unresolved. Persistent astrocytic chromatin remodeling may represent adaptive memory, maladaptive priming, accelerated epigenetic aging, or a combination of these states. Defining how injury-induced epigenetic changes intersect with aging pathways will be critical for understanding why TBI increases susceptibility to late-life neurodegeneration and for identifying interventions that restore glial homeostasis.

### Post-traumatic stress disorder (PTSD)

Post-traumatic stress disorder (PTSD) and major depressive disorder (MDD) are among the most common chronic neuropsychiatric sequelae of TBI and contribute substantially to long-term disability and reduced quality of life [122, 123]. Although the mechanisms linking TBI to these disorders remain incompletely understood, accumulating evidence suggests that persistent neuroinflammation, altered stress responsivity, and epigenetic dysregulation may contribute to their development [124]. Given the emerging role of astrocytes in regulating neuroinflammatory and neuroendocrine signaling, astrocyte epigenetic remodeling may represent a mechanistic bridge between acute brain injury and chronic psychiatric outcomes.

The relevance of persistent epigenetic remodeling extends beyond mechanical injury. Traumatic stress alone can induce durable epigenetic alterations in the brain [125, 126]. PTSD is associated with durable epigenetic alterations affecting genes regulating stress responsivity and synaptic function [127, 128]. DNA methylation may be of clinical utility in the diagnosis and treatment of PTSD [129, 130]. Astrocytes express high levels of glucocorticoid receptors and undergo sustained epigenetic remodeling in response to chronic stress [131, 132]. Consistent with astrocyte involvement in PTSD biology, recent human studies have identified associations between PTSD severity, GFAP-related genetic and epigenetic loci, and altered circulating GFAP levels [133]. The direction of GFAP change differs between PTSD and TBI contexts: suppressed in the former, elevated in the latter. This divergence may reflect distinct forms of astrocyte adaptation or dysfunction and underscore the sensitivity of astrocyte biology to both mechanical and neuroendocrine forms of stress.

The functional significance of this astrocyte-specific chromatin remodeling has been demonstrated in a major depressive disorder model. The authors identified ZBTB7A as a key transcription factor driving stress-induced chromatin remodeling selectively in astrocytes of the orbitofrontal cortex [134]. Astrocytic ZBTB7A induction was both necessary and sufficient to produce stress susceptibility and behavioral deficits in rodents, shifting astrocyte gene expression away from homeostatic and metabolic programs toward inflammatory activity and compromising glutamatergic regulation. Notably, cell-type-specific chromatin accessibility profiling of human postmortem tissue mapped major depression-linked epigenomic features to astrocytes rather than neurons [134], providing cross-species validation of astrocyte chromatin remodeling as a primary locus of stress-related pathology.

PTSD and MDD are common long-term consequences of TBI, and accumulating evidence suggests that their epigenetic signatures partially converge with pathways altered after brain injury, including neuroinflammation, glucocorticoid signaling, and synaptic dysfunction [135, 136]. This overlap support the possibility that astrocytes integrate mechanical injury and neuroendocrine stress signals through shared epigenetic mechanisms, potentially promoting persistent maladaptive responses. Consistent with a functional role for epigenetic dysregulation in trauma-related psychiatric disorders, pharmacological modulation of chromatin remodeling has shown therapeutic benefit in preclinical PTSD models [137]. Whether similar epigenetic interventions can ameliorate PTSD- or depression-like symptoms following TBI, particularly through modulation of astrocyte-specific programs, remains largely unexplored.

Taken together, emerging evidence indicates that epigenetic alterations in reactive astrocytes can establish maladaptive states that drive multiple facets of chronic TBI pathophysiology. Although the precise persistence and causality of these epigenetic states in human TBI remain under investigation, their convergent functional impact points to a shared epigenetic regulatory node. The convergence of epigenetically regulated astrocyte dysfunction across inflammatory, metabolic, vascular, and neurodegenerative domains suggests that chromatin-level modulation may represent an upstream therapeutic strategy.

## Therapeutic targeting of astrocyte epigenetic states

The reversibility of epigenetic modifications positions chromatin regulation as an attractive therapeutic target in TBI [21]. Preclinical studies show that modulating DNA methylation, histone acetylation, and non-coding RNA networks can attenuate inflammation, preserve BBB integrity, and promote functional recovery (Table 1). Importantly, these interventions may act upstream of multiple downstream signaling cascades, offering network-level modulation rather than single-pathway suppression [21]. However, most current approaches lack astrocyte specificity and precise temporal control, underscoring the need for refined strategies.

### Targeting DNA methylation

DNMT inhibitors have demonstrated efficacy in TBI injury models to normalize methylation levels, reducing oxidative stress and attenuating neuronal loss [29]. Selective inhibition of DNMT3A attenuates pathological silencing of astrocyte support genes [16], while modulation of TET3 activity restrains inflammatory gene programs driven by hydroxymethylation at regulatory loci [138].

Because DNA methylation regulates genome-wide transcriptional stability, global inhibition carries off-target risk and translational limitations [139, 140]. Thus, while proof-of-concept efficacy is established in preclinical models, therapeutic application will require greater locus and cell-type specificity.

### Histone modification interventions

HDAC inhibitors represent the most extensively studied epigenetic intervention in TBI. Bromodomain and extra-terminal (BET) proteins function as epigenetic “readers” that recognize and bind acetylated histones [141]. Both HDAC inhibitors and BET inhibitors have therefore been explored as strategies to restore histone acetylation balance after injury. HDAC inhibitors, such as valproic acid [37, 142], sodium butyrate [143], and suberoylanilide hydroxamic acid (also known as Vorinostat) [38], reduce lesion volume, suppress inflammatory cytokines, preserve synaptic proteins, and improve behavioral recovery in rodent models. BET inhibitors similarly modulate inflammatory and plasticity-associated gene programs [42]. Mechanistically, increased histone acetylation at neuroprotective gene loci upregulates neurotrophic factors such as BDNF, attenuates proinflammatory NF-κB signaling in astrocytes, and reduces glial scarring. Class I HDAC inhibitors show particular benefit in preserving neurotrophic support, while BET inhibitors modulate inflammatory and plasticity-associated gene programs. Collectively, these findings suggest that injury-induced epigenetic remodeling contributes to secondary injury progression and that restoring histone acetylation balance represents a viable therapeutic principle. Yet most currently available agents remain systemically active and lack astrocyte selectivity.

### Non-coding RNA-based therapies

MicroRNAs and long non-coding RNAs represent therapeutically tractable targets given their cell-type-enriched expression patterns [144, 145]. miRNA antagomirs or mimics can be designed to inhibit proinflammatory microRNAs such as miR-155, one of the best-characterized inflammatory miRNAs implicated in TBI, attenuating NF-κB and JAK-STAT signaling and reducing cytokine release from reactive astrocytes [146]. While MiR155 expression is observed in neurons, astrocytes and microglia in the perilesional cortex of rats after TBI, it is most prominently expressed in reactive astrocytes in the human perilesional cortex [49].

Long non-coding RNAs participate in astrocyte-microglia communication. Astrocyte-derived exosomal lncRNAs are reported to exert anti-inflammatory effects on TBI-induced microglial reactivity and ameliorate cognitive function deficiency [67]. The cell-type-enriched expression of many non-coding RNAs may confer greater specificity than global chromatin-modifying strategies [147, 148]. However, efficient and targeted CNS delivery remains a major translational barrier. Viral vectors [149], lipid nanoparticles [150], and exosome-based systems [151] have demonstrated feasibility in preclinical models, but require optimization for safety, specificity, and scalable clinical translation. Despite these barriers, RNA-based strategies offer flexible and potentially reversible modulation of astrocytic gene networks.

### Limitations of current epigenetic interventions

A major limitation of current epigenetic therapeutic strategies is the lack of cell-type specificity. Systemic inhibition of HDACs, DNMTs, BET proteins, or HATs alters chromatin architecture simultaneously in neurons, astrocytes, microglia, oligodendrocytes, endothelial cells, and peripheral tissues, obscuring the cellular origin of therapeutic benefit. Moreover, astrocytes themselves exhibit substantial functional heterogeneity across brain regions and injury phases [152]. Therefore, global chromatin modulation may suppress maladaptive inflammatory programs while inadvertently disrupting reparative or homeostatic functions. Compounding this complexity, the biological consequences of histone acetylation and DNA methylation changes are highly phase dependent. Interventions beneficial during the acute stage may prove ineffective or deleterious in chronic stages. Moreover, many agents demonstrate narrow therapeutic windows [153]. Thus, temporal precision is as important as molecular specificity in designing effective epigenetic interventions.

Translation to the clinical setting introduces additional challenges. Species differences in glial biology and epigenetic landscape organization limit direct extrapolation from rodent models to humans [154]. The dynamic and evolving nature of TBI pathophysiology further necessitates phase-matched intervention strategies, as indiscriminate or poorly timed chromatin modulation may exacerbate secondary injury cascades.

In summary, existing epigenetic interventions establish proof of concept but fall short of the precision required for clinical translation. As summarized in Table 2, current approaches vary substantially in their specificity, reversibility, delivery feasibility, and translational readiness, highlighting the need for more precise and cell-type-targeted therapeutic strategies. Closing the gaps in cell-type specificity, temporal control, and human validation will be essential for moving these strategies from the bench toward meaningful therapeutic application.

**Table 2:** Comparative overview of emerging epigenetic therapeutic strategies for TBI

Strategy	Potential Strengths	Specificity	Reversibility	CNS Delivery	Clinical Readiness	Major Limitations
DNMT/TET modulation	Targets persistent DNA modification states; potential to reverse long-term epigenetic dysregulation	Low–Moderate	Moderate	Feasible	Preclinical	Broad genomic effects, limited locus specificity
HDAC/BET inhibition	Most extensively studied; multiple compounds already clinically available for other indications	Low–Moderate	High	Feasible	Preclinical for TBI	Limited cell-type specificity, off-target effects
Non-coding RNA therapeutics	Potentially high target specificity and reversibility; cell-type-enriched expression patterns	Moderate–High	High	Challenging	Early preclinical	Delivery, stability, and target engagement
CRISPR-based epigenome editing	High precision targeting of defined regulatory loci	High	Platform dependent	Challenging	Experimental	Delivery, safety, and long-term effects; reversibility depends on editing strategy
Astrocyte-targeted delivery platforms	May improve cell-type specificity and reduce off-target effects	Moderate–High (vector and cargo dependent)	Cargo dependent	Major challenge	Experimental	Targeting efficiency, vector limitations, and translational scalability

## Future directions

**Fig. 3:**
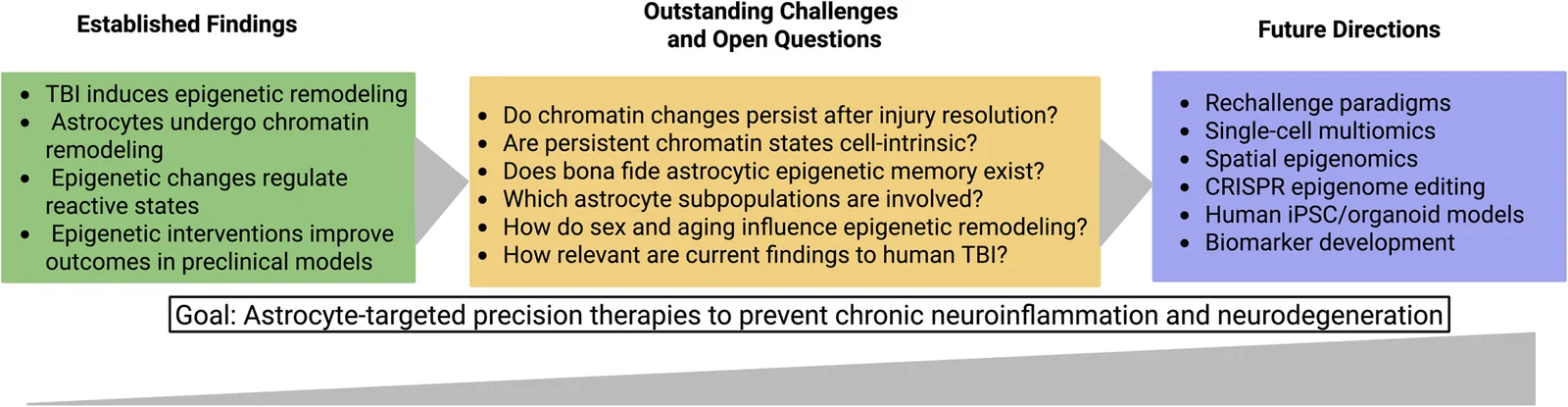
Current understanding, unresolved questions, and future directions in astrocyte epigenetic remodeling after TBI. Established findings (left) indicate that TBI induces epigenetic remodeling in astrocytes, that these changes contribute to reactive state transitions, and that epigenetic interventions improve outcomes in preclinical models. Outstanding challenges (center) include determining whether injury-induced chromatin changes persist after injury resolution, whether persistent chromatin states are cell-intrinsic, and whether bona fide astrocytic epigenetic memory exists. Additional open questions concern the astrocyte subpopulations involved, the influence of sex and aging on epigenetic remodeling, and the relevance of current findings to human TBI. Future directions (right) include rechallenge paradigms, single-cell multiomics, spatial epigenomics, CRISPR-based epigenome editing, human iPSC and organoid models, and epigenetic biomarker development. Together, these approaches may enable the development of astrocyte-targeted precision therapies to prevent chronic neuroinflammation and neurodegeneration after TBI. Created with BioRender

Advancing astrocyte-centered epigenetic therapies for TBI will require integration of high-resolution profiling, precision editing tools, and translational biomarker development. The next phase of the field must move from descriptive epigenomics toward mechanistic, cell-type-specific intervention. The major established findings, outstanding challenges, and future directions discussed in this section are summarized in Fig. 3.

### From bulk to single-cell and spatial epigenomics

Most epigenetic studies in TBI have relied on bulk tissue analyses that obscure astrocyte heterogeneity and regional specialization. Single-cell and single-nucleus RNA-sequencing, ATAC-seq, and multi-omic platforms now allow simultaneous profiling of chromatin accessibility, DNA methylation, and transcriptomes at cellular resolution [155, 156]. These approaches will allow identification of transcription networks and chromatin states unique to specific astrocyte subpopulations/states. They will also help with moving beyond the oversimplified binary of neurotoxic versus neuroprotective astrocytes and support a higher-resolution classification of functionally distinct states [14, 157].

Integration with spatial transcriptomics and spatial epigenomics will be important for mapping reactive astrocyte states across injury cores and perilesional regions and linking epigenetic states to local microenvironments [158–160]. Longitudinal sampling across acute, subacute, and chronic stages will further help distinguish stable epigenetic remodeling from reversible activation states.

### Deciphering astrocytic epigenetic memory

Whether astrocytes acquire durable epigenetic memory after TBI that shapes responses to subsequent insults remains a central unanswered question. The importance of rigorous interpretation is illustrated by a recent debate in the EAE literature, in which O'Dea and Liddelow challenged the astrocytic memory signature reported by Lee et al., arguing that it reflected immune cell contamination rather than an astrocyte-intrinsic phenomenon [161]; the original authors subsequently published a detailed rebuttal [162]. The exchange underscores a fundamental challenge: persistent transcriptional activation does not establish memory, and may instead reflect ongoing inflammation, incomplete resolution, or technical confounds—distinctions particularly critical in TBI, where chronic microenvironmental alterations are pervasive.

Convincing demonstration of epigenetic memory requires four criteria: (1) an injury-dependent chromatin remodeling event; (2) persistence after acute injury resolution; (3) cell-intrinsic stability independent of continuous inflammatory signaling; and (4) an altered response upon secondary challenge. Current TBI studies support the first criterion with partial evidence of persistence, but cell autonomy and rechallenge responses remain largely unaddressed. Washout and rechallenge paradigms are therefore necessary to distinguish durable memory from chronic activation.

Disentangling cell-intrinsic memory from sustained environmental reinforcement is equally important, as ongoing cytokine signaling, microglial activation, and matrix remodeling could maintain chromatin accessibility without self-sustaining epigenetic mechanisms [100]. Stronger evidence would require persistence of injury-associated enhancer states following isolation under cytokine-free conditions or transplantation into naïve environments.

Overlap with senescence- or stress-induced remodeling represents an additional confounder [163, 164]; true memory should exhibit stimulus specificity and functional recall, whereas senescence-related changes manifest as broader transcriptional instability. Longitudinal epigenomic profiling combined with functional restimulation assays will be required to determine whether astrocyte epigenetic memory after TBI represents a discrete and therapeutically tractable biological state.

Repetitive mild TBI offers an especially relevant context for testing this hypothesis. A first mild injury may establish persistent astrocytic chromatin alterations that prime inflammatory, metabolic, or neurovascular pathways, modifying responses to later insults. Determining whether a single mild TBI induces stable, cell-intrinsic epigenetic changes that alter astrocyte behavior upon reinjury would provide a rigorous test of the epigenetic memory hypothesis and may help explain cumulative effects observed after repetitive head trauma.

Genomic imprinting represents an underexplored dimension of TBI epigenetics. Because many imprinted genes regulate synaptic plasticity, cognition, and behavior [165], disruption of imprinting control regions could influence long-term neurological outcomes after injury. Whether TBI alters imprinting control regions or induces persistent dysregulation of imprinted genes in astrocytes remains unknown and warrants further investigation.

### Functional validation of astrocytic epigenetic remodeling

Most studies describing injury-induced chromatin remodeling in astrocytes remain correlative. Persistent H3K27ac enrichment, altered chromatin accessibility, or DNA methylation changes at inflammatory loci are consistent with epigenetic reprogramming but do not establish functional necessity.

CRISPR-dCas9 epigenetic editors fused to methyltransferases, demethylases, or acetyltransferases enable precise manipulation of candidate regulatory elements without altering DNA sequence [166]. Astrocyte-restricted deletion of epigenetic regulators and enhancer perturbation approaches [73] can test whether defined chromatin states drive inflammatory priming, metabolic dysfunction, or repair programs. Rechallenge paradigms following targeted editing will determine whether persistent chromatin states alter responses to subsequent stimuli. Such studies are essential for establishing causal links between epigenetic remodeling and astrocyte function after TBI.

### Precision epigenetic therapeutics

Therapeutic translation requires stringent specificity. Systemic inhibition of DNMTs or HDACs affects multiple CNS cell types and peripheral tissues, risking disruption of adaptive programs.

CRISPR-based epigenetic editors offer precise and potentially reversible modulation without permanent DNA alterations [166]. Astrocyte-specific delivery platforms—including GFAP-promoter-driven vectors [167], nanoparticle carriers, and exosome-based systems [149–151]—limit off-target effects, while isoform-selective inhibitors targeting discrete HDACs [58], DNMTs [168], or inflammatory transcriptional regulators provide complementary temporal control. Therapeutic design must account for astrocyte heterogeneity; reactive astrocytes represent context-dependent states rather than a uniform pathological entity [14, 157], and indiscriminate suppression may impair repair functions. Integration of single-cell epigenomics with targeted delivery technologies will be necessary to identify subtype-specific regulatory nodes suitable for precise and safe intervention.

### Sex as a biological variable in astrocyte epigenetic remodeling

An additional knowledge gap concerns the influence of biological sex on astrocyte epigenetic remodeling after TBI. Injury-induced molecular responses may differ between males and females as shown by the sex differences in TBI incidence, symptom burden, recovery trajectories, and neurodegenerative risk [169–171]. Sex hormones can directly influence DNA methylation, histone modifications, chromatin accessibility, and inflammatory gene expression programs, and astrocytes exhibit sex-dependent differences in reactivity and neuroprotective functions [172]. However, studies specifically examining astrocyte epigenetic regulation after TBI remain limited. Whether biological sex affects the putative astrocytic epigenetic memory is unknown. Future studies incorporating both sexes and integrating single-cell epigenomic approaches will be essential to address this question.

### Biomarker development

Epigenetic signatures in glial-derived exosomes and cell-free DNA may provide accessible biomarkers of astrocyte state and help predict TBI severity, recovery, and neurodegenerative risk [173–175]. Methylation signatures at astrocyte-associated regulatory loci show particular promise [70]. Integration of epigenetic biomarkers with imaging and proteomic measures may improve patient stratification and outcome prediction [133, 176].

### Human-relevant models and translational validation

Much of our current mechanistic understanding derives from animal models that do not fully capture the complexity of human TBI [154]. The development of effective diagnostic and therapeutic strategies for TBI requires a detailed understanding of human neurophysiology [155]. Multiomic profiling of postmortem human TBI tissue [155, 177], human iPSC-derived astrocytes [178], and brain organoid systems [179, 180] provide complementary platforms for validation.

In summary, advancing this field requires moving beyond descriptive epigenetic profiling toward mechanistic, cell-type-specific, and translationally grounded approaches. Astrocytic epigenetic memory after TBI remains plausible but unproven. In light of critiques from EAE studies, future research must demonstrate cell autonomy, lineage continuity, stimulus specificity, and functional recall. Integrating single-cell epigenomics, functional perturbation studies, metabolic insights, and human validation will enable rigorous testing of the memory hypothesis. This will also clarify how astrocytic chromatin remodeling shapes TBI progression and help identify therapeutic targets to improve long-term outcomes.

## Conclusion

TBI initiates a dynamic secondary injury cascade in which astrocytes shift from homeostatic regulators to key contributors towards neurological outcomes [97]. This review positions epigenetic remodeling as the molecular substrate of that shift. Coordinated alterations in DNA methylation, histone modifications, chromatin accessibility, and non-coding RNA networks may stabilize injury-induced transcriptional programs and convert transient mechanical signals into persistent astrocytic states.

These chromatin-level changes provide a unifying mechanism for the dual nature of reactive astrocytes. The same epigenetic architecture can sustain neuroinflammation or alternatively facilitate repair and neuroplasticity. The concept of astrocytic epigenetic memory further extends this model. It suggests that prior injury can be embedded within chromatin configuration. Importantly, this imprint shapes long-term responsiveness and potentially contributes to chronic neurodegenerative diseases.

Crucially, epigenetic regulation is reversible. Preclinical modulation of methylation enzymes, histone-modifying complexes, and regulatory RNA networks suggests that astrocyte reactivity is modifiable. Although challenges in cell-type specificity, drug delivery, and temporal precision remain, these studies establish proof-of-concept that chromatin-targeted interventions can reshape the secondary injury cascade.

Emerging technologies, including single-cell multi-omics, spatial epigenomics, and locus-specific epigenome editing, now enable mechanistic resolution at cellular and genomic scales that were previously inaccessible. Coupled with biomarker development and human validation through iPSC-derived models and postmortem tissue profiling, these tools position the field to move from descriptive profiling toward precision modulation of defined astrocyte states.

Several conclusions emerge from the current literature. First, epigenetic remodeling is a robust and reproducible consequence of TBI, encompassing DNA methylation, histone modifications, chromatin accessibility, and non-coding RNA networks. Second, astrocytes are increasingly recognized as important targets and mediators of these changes. Growing evidence links epigenetic remodeling to neuroinflammation, metabolic dysfunction, neurovascular impairment, and increased vulnerability to neurodegeneration. However, major questions remain unresolved. In particular, the persistence, cell autonomy, and functional consequences of injury-induced epigenetic remodeling in astrocytes are incompletely understood. It remains unclear whether TBI establishes bona fide memory-like states in astrocytes that alter responses to subsequent challenges. Addressing these questions will require longitudinal multi-omic profiling, spatially resolved analyses, cell-type-specific epigenetic perturbation, and rechallenge paradigms. Resolving these issues will be essential for determining whether astrocyte epigenetic remodeling represents not only a marker of injury, but also a mechanistic driver of chronic pathology and a tractable therapeutic target for the long-term consequences of TBI.

## Data Availability

No datasets were generated or analysed during the current study.
